# Turtle Dorsal Cortex Pyramidal Neurons Comprise Two Distinct Cell Types with Indistinguishable Visual Responses

**DOI:** 10.1371/journal.pone.0144012

**Published:** 2015-12-03

**Authors:** Thomas Crockett, Nathaniel Wright, Stephen Thornquist, Michael Ariel, Ralf Wessel

**Affiliations:** 1 Department of Physics, Washington University in St. Louis, St. Louis, Missouri, United States of America; 2 Department of Pharmacology and Physiology, Saint Louis University School of Medicine, St. Louis, Missouri, United States of America; McGill University, CANADA

## Abstract

A detailed inventory of the constituent pieces in cerebral cortex is considered essential to understand the principles underlying cortical signal processing. Specifically, the search for pyramidal neuron subtypes is partly motivated by the hypothesis that a subtype-specific division of labor could create a rich substrate for computation. On the other hand, the extreme integration of individual neurons into the collective cortical circuit promotes the hypothesis that cellular individuality represents a smaller computational role within the context of the larger network. These competing hypotheses raise the important question to what extent the computational function of a neuron is determined by its individual type or by its circuit connections. We created electrophysiological profiles from pyramidal neurons within the sole cellular layer of turtle visual cortex by measuring responses to current injection using whole-cell recordings. A blind clustering algorithm applied to these data revealed the presence of two principle types of pyramidal neurons. Brief diffuse light flashes triggered membrane potential fluctuations in those same cortical neurons. The apparently network driven variability of the visual responses concealed the existence of subtypes. In conclusion, our results support the notion that the importance of diverse intrinsic physiological properties is minimized when neurons are embedded in a synaptic recurrent network.

## Introduction

**Fig 1:**
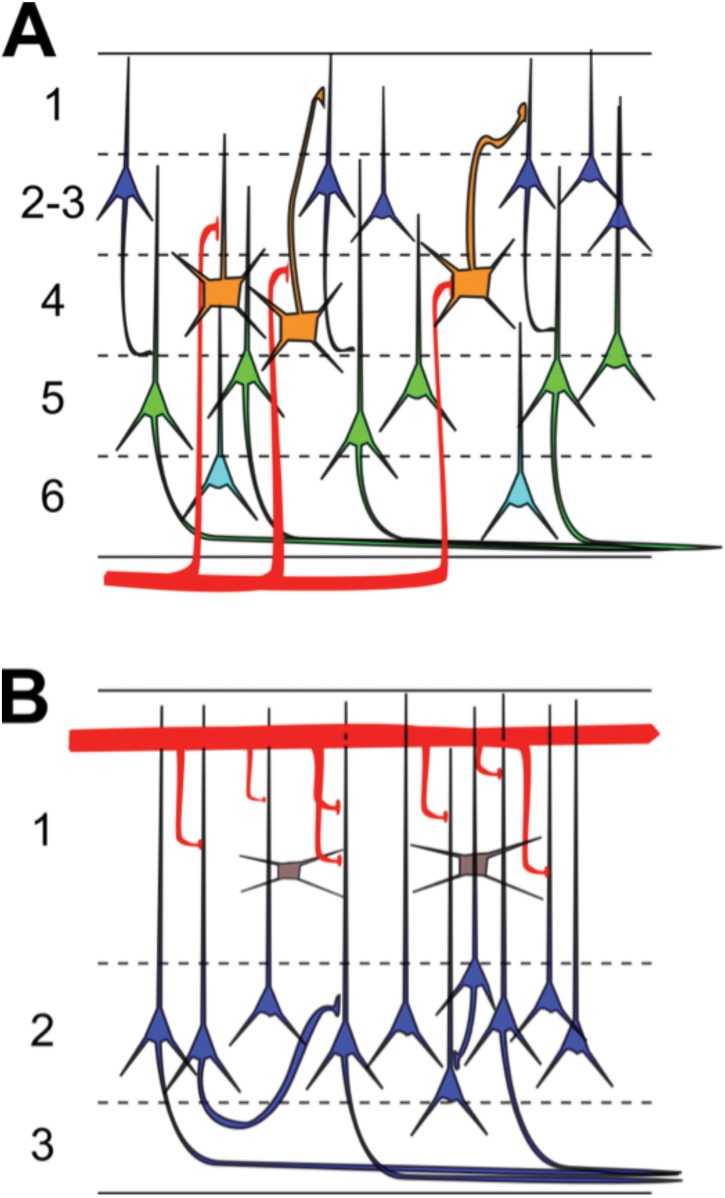
Basic microcircuit of neocortex and turtle dorsal cortex. (A) The neocortex consists of 6 layers with multiple types of pyramidal neurons (color) and thalamic inputs (red) terminating in spatially restricted regions. For clarity, interneurons are omitted in this schematic diagram. (B) The turtle dorsal cortex consists of one cellular layer (#2) of densely packed pyramidal neurons (blue), sandwiched between two neuropil layers (#1 and 3) that are densely packed with dendrites and axons, and also contain interneurons (grey). Sensory afferents (red) from the lateral geniculate nucleus (LGN) make en-passant synapses in superficial layer 1 on distal segments of pyramidal neuron dendrites and on superficial inhibitory interneurons.

Cortical pyramidal neuron subtype classification has become an area of intense research in neuroscience [1]. Cortical pyramidal neurons display a vast diversity of properties in numerous dimensions, including morphology, electrophysiology, gene expression, connectivity, and axonal projections [2–5]. Many of these properties covary, indicating that the heterogeneity found in pyramidal neurons is not due to random events but instead due to their separation into specific cellular subtypes [6] that is choreographed by transcriptional regulation during neuronal development [7]. Generating a census of pyramidal neuron subtypes is thought fundamental to the accurate observation and manipulation of brain activity [8] and to the development of cell-type and circuit-specific therapies to treat brain disorders [9]. As a case in point, the laminar organization of pyramidal neurons in neocortex (Fig 1A) plays a key role in the processing of visual inputs, as indicated by layer and cell-type specificity of sensory responses [10–14].

The search for pyramidal neuron subtypes is particularly significant in the trilaminar allocortex [15], which contains a single layer of densely packed somata of pyramidal neurons sandwiched between layers filled with dendrites, axons, and a few scattered interneurons (Fig 1B). In part because of its ancestral position in evolutionary history [16], information about the allocortex is believed to facilitate the investigation of the neocortex [17, 18]. Three prominent examples of allocortex are the mammalian piriform cortex [19] and hippocampus [20], and the reptilian dorsal cortex ([21]). Based on morphological and intrinsic electrophysiological properties, two classes of pyramidal neurons were classified in mouse piriform cortex [22, 23] and the CA1 and subiculum regions of rat hippocampus [24]. Less is known about the reptilian dorsal cortex, which holds a strategic position among the examples of allocortex. It receives input from lateral geniculate nucleus (LGN) [25, 26] and thus exemplifies a trilaminar visual cortex [21, 27] that processes information from a well-defined spatio-temporal-chromatic visual input space. Information about pyramidal neuron subtypes in the trilaminar dorsal cortex is limited. Variations of properties among pyramidal neurons in the dorsal cortex of turtle [28] include firing patterns [29], axonal projection targets [30], and molecular markers [31]. It is not known however, whether the variation of properties reflects the existence of pyramidal neuron subtypes or the broad distribution of properties in one neuron type.

**Fig 2:**
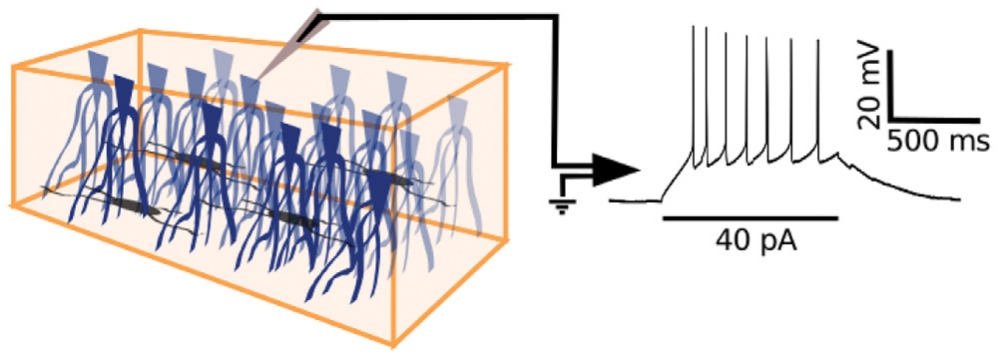
Whole-cell recordings from pyramidal neurons in turtle visual cortex. Schematic diagram of an isolated piece of turtle dorsal cortex (left panel) with the ventricular side up and containing pyramidal neurons (blue) and interneurons (grey). A whole-cell recording of the pyramidal neuron membrane potential in response to current injection (right panel) is obtained with a patch electrode (grey triangle) that is positioned at the pyramidal neuron soma under visual guidance with DIC optics.

Here, we investigate pyramidal neurons within the single layer of densely packed somata of turtle dorsal cortex (Fig 2) and uncover the presence of two main electrophysiological types, however with highly fluctuating and indistinguishable responses to visual stimulation of the retina.

## Materials and Methods

### *Ex vivo* cortex preparation, visual stimulation, and intracellular recording

Procedures used in this study were approved by Washington University’s Institutional Animal Care and Use Committee and conform to the guidelines of the National Institutes of Health on the Care and Use of Laboratory Animals. Red-eared slider turtles (*Trachemys scripta elegans*, 150–200 g weight, 12–15 cm carapace length, of either sex. Niles Biological Inc, Sacramento, CA, USA) were used in this study. Following anesthesia (intravenous propofol 10 mg/kg) and rapid decapitation by a guillotine, we surgically removed the brain, optic nerves, and eyes from the cranium as described earlier [29, 32–34]. In brief, during surgery we cut the conjunctiva and extraocular muscles to free the eyes from their orbits. After removing the brain from the skull, we cut rostro-caudally along the medial cortex, starting at the ventricle of the olfactory bulb. This cut preserves the normal afferent input of the visual cortical area, because the geniculocortical pathway traverses the lateral cortical wall within the lateral forebrain bundle [26]. Subsequently, two medio-lateral cuts to the telencephalon at its anterior and posterior ends prepared for unfolding of the hemisphere and exposing the ventricular surface. For the isolated-cortex preparation, a slab of the cortex was separated from the rest of the brain by cutting along the dorsal ventricular ridge. For the eye-attached whole-brain preparation, the anterior half of the contralateral eye was removed and the vitreous was drained to expose the retina in an eye-cup; the ipsilateral eye was removed. The preparation (cortex slab or eye-attached whole-brain) was transferred to the recording chamber (RC-27LD, Warner Instruments) positioned on an air table and under a fixed-stage upright fluorescent microscope (BX-51WI, Olympus) equipped with differential interference contrast (DIC) optics. The unfolded cortex with the ventricular side up was pinned with short pieces of tungsten wires (25 μm diameter) to a Sylgard (Dow Corning) anchor at the periphery of the recording chamber. The eye-cup, brain, and/or cortex were continuously perfused (2 mL/min) with artificial cerebrospinal fluid (in mM; 85 NaCl, 2 KCl, 2 MgCl2, 45 Na HCO3, 20 D glucose, and 3 CaCl2 bubbled with 95% O2 and 5% CO2), adjusted to pH 7.4 at room temperature. For diffuse whole-field visual stimulation of the retina, a red light emitting diode (LED) was positioned 2 cm above the eye cup. Timed brief flashes of 10 ms duration were presented with at least thirty seconds between flashes. Approximately 2–3 hrs passed between induction of anesthesia and the start of the experimental recordings. Whole-cell recordings from neurons within the cellular layer of visual cortex were obtained with visually guided (DIC optics) patching using pipettes (4–8 MΩ) pulled from borosilicate glass and filled with a standard electrode solution (in mM, 124 KMeSO_4_, 2.3 CaCl_2_-2H_2_0, 1.2 MgCl_2_, 10 HEPES, 5 EGTA) with 0.04% biocytin for intracellular labeling. Current clamp recordings were made at room temperature (21–24°C) using an AxoClamp 2B (Axon Instruments) amplifier, digitized with an acquisition board (National Instruments PCI-MIO-16E-4) and controlled using custom LabVIEW software. In turtle visual cortex, spike train differences between pyramidal neurons and interneurons have been reported [28, 35]. In addition, we are confident that the vast majority of our neurons are indeed pyramidal for the following three reasons. However, we are confident that the vast majority of our neurons are indeed pyramidal. First, neurons were selected from within the densely packed cellular layer, which is clearly distinguishable using DIC optics due to the striking increase in the density of neurons versus the less populous layers 1 and 3. Second, pyramidal neurons account for approximately 80–90% of neurons in the dorsal cortex and are by far the most numerous neurons in layer 2 [21]. Third, histological spot checks of a third of the neurons in our data set revealed no morphological evidence for interneurons. Therefore, we assume that any contribution of interneurons to the dataset is negligible and we refer to the dataset of recorded neurons as pyramidal neurons. A recorded neuron was accepted in the database when the membrane potential was more negative than -40 mV and the action potential amplitude was larger than 25 mV.

### Electrophysiological analysis

Following five minutes of recording spontaneous membrane potential fluctuations, 14 electrophysiological parameters were obtained for subsequent pyramidal neuron type analysis: (1) Resting membrane potential (V_rest_) was measured and current was injected through the recording electrode for 1 second duration with at least 2 seconds of wait time between trials, starting at -70 pA and increasing by 10 pA each trial until a spike was elicited [36–38]. Although the pyramidal neurons were studied within their endogenous cortical circuits, there was no evidence from these recordings that synaptic activity was being modulated by the injected current pulses. We presume, therefore, that the 14 electrophysiological parameters only reflect the intrinsic nature of the recorded neuron. (2) Rheobase current (I_r_) was defined as the lowest current for which an action potential was elicited in three consecutive trials [39, 40]. Single-spike parameters (3–8) were determined by averaging at least three trials of rheobase current injection. (3) Action potential voltage threshold (V_th_) was determined as the point of maximum inflection where the third derivative of the voltage is maximized [37, 40, 41]. (4) Action potential amplitude (AP Height) was measured from voltage threshold to the peak of the action potential [42–45]. Action potential duration was measured both (5) halfway between threshold and peak (W_AP_) [38, 45, 46] and (6) at threshold (W_AP,thresh_) [37, 39, 47]. It should be noted that this definition of “action potential width at threshold (W_AP,thresh_)” can lead to spurious results when applied to neurons with complex and long-lasting depolarization above threshold following the first action potential [48]. (7) The action potential fall rate (Min(dV/dt)) was measured as the maximum downslope in the falling phase of the spike [40, 46, 47]. (8) The time to peak of the afterhyperpolarization (L_AHP_) was measured as the time elapsed between crossing the threshold voltage in the falling phase of the action potential to the peak of the afterhyperpolarization [39, 42]. This peak was defined as the post-spike voltage trough. (9) Input resistance (IR) was determined by examining the membrane voltage drop in response to 1-s hyperpolarizing current pulses [38, 43–45]. (10) The membrane time constant (t_m_) was determined by fitting an exponential function (Kaleidagraph, Synergy Software, Reading, PA) to voltage traces in response to hyperpolarizing current injection [36, 40, 47]. Spike train parameters (11–14) were measured and averaged for several trials where multiple spikes were elicited in response to depolarizing 1-s current pulses. (11) Action potential duration increase (W_AP Inc._) is (*D*_2_ − *D*_1_)/*D*_2_ where *D*_1_ and *D*_2_ are the durations at half-max (5) of the first and second spikes, respectively, after beginning depolarizing current injection [43, 45, 49]. (12) Action potential amplitude decrement (V_peak Dec._) is (*A*_1_ − *A*_2_)/*A*_1_ where *A*_1_ and *A*_2_ are the amplitudes (4) of those spikes [43, 45]. (13) Action potential frequency adaptation ratio (AP FAR) is the ratio of the first interspike interval (ISI) to the average of the last three interspike intervals [37, 50, 51]. (14) Interspike interval variability (ISI Var) during rhythmic firing is the variance of the interspike intervals over the current injection interval [39]. Neurons that never elicited more than one spike were assigned an AP duration increase of 200, an AP amplitude reduction of 100, an adaptation ratio of 0, and an ISI variability of 0.

### Unsupervised clustering

To classify neurons, unsupervised clustering using Ward’s linkage method was utilized [52]. In this algorithm, neurons begin as individual points in a 14-dimensional parameter space and are grouped in a bottom-up fashion. To ensure that equal weight be given to each of them, parameters were standardized with a mean of 0 and a standard deviation of 1 [38] prior to analysis. At each step, the neurons or clusters of neurons with minimum between-cluster distance (least Euclidean distance) are merged into a larger cluster. Operationally this is equivalent to finding the pair of clusters that leads to minimum increase in total within-cluster variance after merging. The result of this recursive algorithm is visualized on a dendrogram (tree diagram) as a horizontal bar joining the neurons drawn at a height corresponding to the linkage distance–the Euclidean distance between the two merged neurons/clusters in parameter space. In the next step, two more neurons/clusters are merged. Eventually, neurons are closer to neuron pairs or higher clusters than to other individuals and merge with those pairs into even greater population subclusters, or clusters merge with one another. These collections continue merging step by step until all neurons are contained within one supercluster.

Ward’s linkage method generates a dendrogram, but does not provide a rationale at what height to cut the dendrogram into groups and thus cannot inform about an absolute number of clusters. We determined the number of putative pyramidal neuron subtypes among the dataset by dividing the clustering tree into higher-order clusters as suggested by the Thorndike procedure [43, 45, 53] and validated by silhouette analysis [54, 55].

Silhouette analysis quantifies the likelihood that a neuron should be a member of the cluster it was placed in by comparing the mean distance in parameter space between the neuron and its intracluster companions, *a*_*i*_ and the mean distance between it and those neurons in the next closest cluster *b*_*i*_ [54, 55] This comparison is normalized by the maximum of the two averages, giving a neuron silhouette value of $S_{i}\;=\;\frac{\left(b_{i}\;-\;a_{i}\right)}{max(a_{i};b_{i})}$, where *S*_*i*_ is strictly bound by −1 <*S*_*i*_<1. A negative value suggests a potential misclassification, since the neuron in question is then closer in parameter space to the members of a different cluster of neurons than to those of the cluster to which it is assigned. The average silhouette value for the clustering is then given by averaging over all of the neurons *S*_*average*_ = 〈*S*_*i*_〉 and thus *S*_*average*_ is also strictly bound, −1< *S*_*average*_ <1.

Complementary to Ward’s clustering, k-means clustering generates clusters in a top-down manner using a predetermined number of k clusters [56]. Starting from a random cluster centroid position, positions are iteratively optimized. Thus suboptimal assignment of neurons to specific clusters is dynamically corrected across iterations. The process is repeated different random initial positions of the k-cluster centroids. In k-means clustering, the number of k clusters must be predetermined and, in this study, was chosen to equal the number of clusters inferred from the Ward’s method. Additional analysis (not shown) was done with higher numbers of clusters.

### Model simulation

The model network consisted of three clusters of neurons separated by type, including two distinct excitatory groups (A and B, 1000 neurons each) and one inhibitory (200 neurons), with reciprocal connectivity. Intracluster (P_in_) and intercluster (P_out_) connection probabilities controlled the likelihood of synaptic connections between neurons. Model neurons were implemented as described earlier [57]. Inhibitory neurons and the excitatory neurons of type A and B differed by their defining parameter values (inhibitory: a = 0.1, b = 2, c = -50, d = 2, C_m_ = 20 μF, R = 100 MΩ; excitatory type A: a = 0.02, b = 0.25, c = -65, d = 0.05, C_m_ = 20 μF, R = 400 MΩ; excitatory type B: a = 0.02, b = 0.2, c = -65, d = 8, C_m_ = 30 μF, R = 250 MΩ). Synaptic currents were simulated as described earlier [58]. Parameters for excitatory currents were V_syn_ = 0 mV, τ_l_ = 0.5 ms, τ_r_ = 0.2 ms, τ_d_ = 1 ms and for inhibitory current were V_syn_ = -70 mV, τ_l_ = 0.5 ms, τ_r_ = 0.5 ms, τ_d_ = 5 ms. Synaptic strength between the possible combinations of external inputs (E), pyramidal neurons (P) and interneurons (I) was parameterized by g_syn_ as follows (in nS): P-to-P, 0.29; P-to-I, 0.3; I-to-P, 3.8; I-to-I, 4.0; E-to-P, 3; E-to-I, 5.2. All neurons received excitatory input in the form uncorrelated Poisson pulse trains at low rate thus generating a baseline level of network activity. The brief external stimulus (mimicking the LGN input caused by a diffuse whole-field flash) to the model network consisted of a 4.5 times increase in the Poisson pulse rate for the duration of 100 ms and exclusively to type A neurons.

## Results

**Fig 3:**
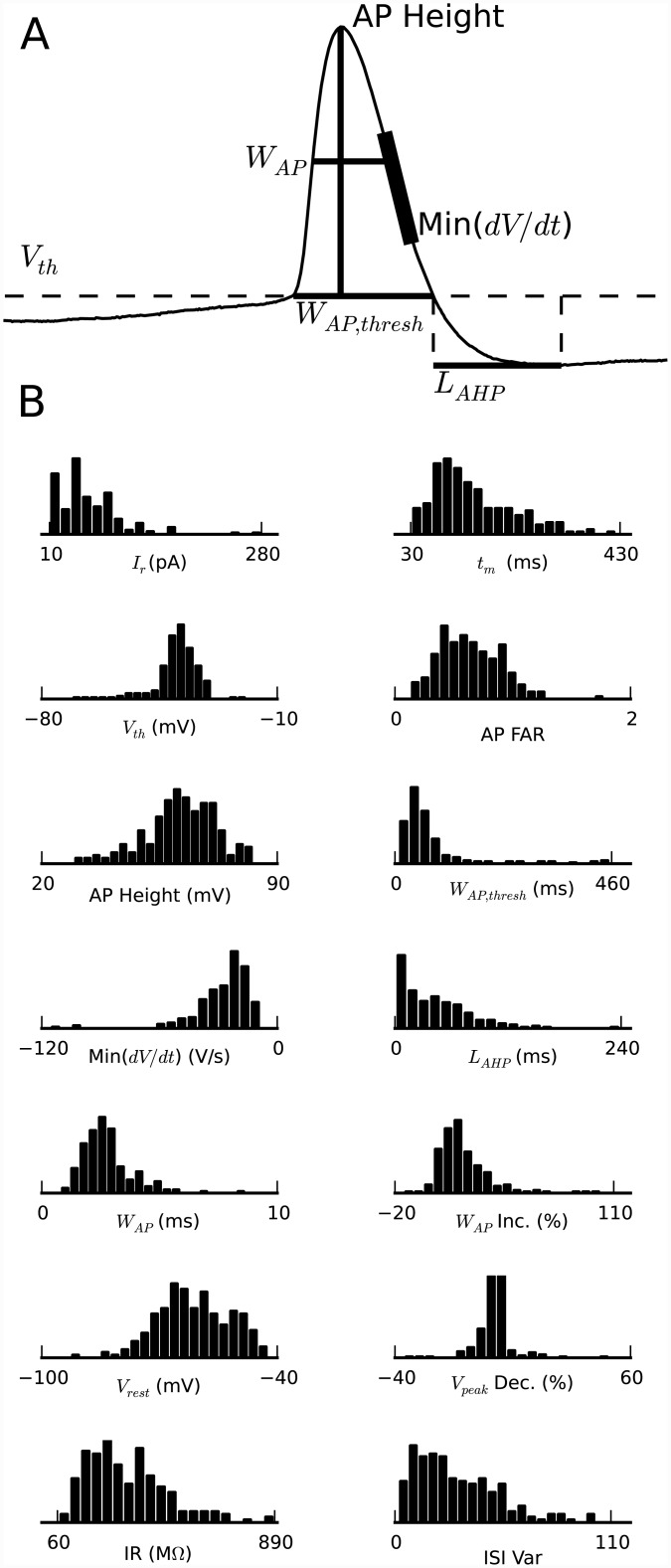
Distribution of electrophysiological properties. (A) Illustration of how a subset of the parameters were measured from the action potential shape of the first action potential in response to somatic current injection: threshold voltage (V_th_), width at threshold (W_AP,thresh_), width at half-max (W_AP_), height, maximum fall rate (Min(dV/dt)), and afterhyperpolarization latency (L_AHP_). (B) Distribution of the measured values for the 14 electrophysiological parameters from membrane potential recordings in response to somatic current injection from 225 pyramidal neurons: rheobase current (I_r_), membrane time constant (t_m_), action potential voltage threshold (V_th_), action potential frequency adaptation ratio (AP FAR), action potential amplitude (AP Height), action potential duration at threshold (W_AP,thresh_), action potential fall rate (Min(dV/dt)), time to peak of the afterhyperpolarization (L_AHP_), action potential duration halfway between threshold and peak (W_AP_), action potential duration increase (W_AP Inc._), resting membrane potential (V_rest_), action potential amplitude decrement (V_peak Dec._), input resistance (IR), interspike interval variability (ISI Var). The apparent deviations from normal distributions suggest that there are discrete groups of pyramidal neurons within this population.

We obtained whole-cell recordings from 225 pyramidal neurons selected under visual guidance with DIC optics from the cellular layer of the turtle visual (dorsal) cortex (Fig 2). The densely packed pyramidal neuron somata within the cellular layer appear indistinguishable by visual inspection. Neurons were selected from a region located central between rostral and caudal dorsal cortex. From the recorded membrane potential responses to somatic current injections (1 s duration; 2 s wait time between trials), we obtained the neurons’ electrophysiological properties, which we quantified with a selected set of 14 parameters (Fig 3A). The parameter set was chosen on the basis of (i) providing distinct, as opposed to redundant, features [46] and (ii) showing variability over the collection of neurons [43, 49]. The resulting distributions of the 14 parameters from the 225 recorded pyramidal neurons were neither multimodal nor Gaussian (Fig 3B), thus indicating the possibility for multiple types of pyramidal neurons within the cellular layer of turtle visual cortex.

### Two main types of pyramidal neurons

**Fig 4:**
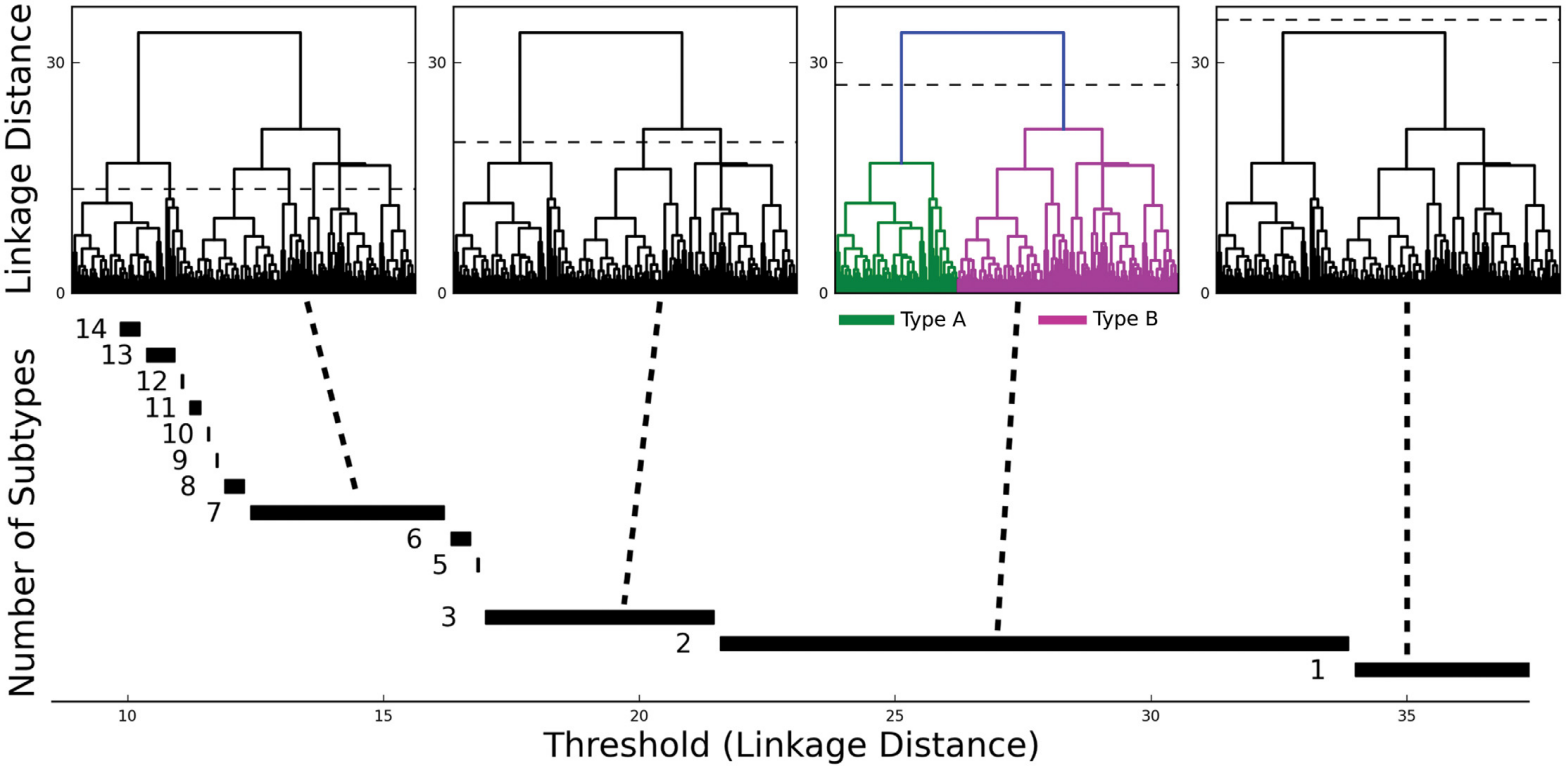
Two main types of pyramidal neurons in turtle dorsal cortex. (TOP) Ward’s unsupervised clustering applied to a sample of 225 pyramidal neurons from turtle dorsal cortex, with each neuron characterized by 14 electrophysiological parameters. The x-axis in each plot represents the individual neurons. The y-axis represents the Euclidean distance between the two merged neurons/clusters in parameter space. Dashed lines in the four identical dendrograms indicate possible threshold choices. The dashed line in the colored dendrogram marks the threshold as suggested by the Thorndike procedure, which indicates two types of pyramidal neurons. (BOTTOM) The number of types increases with decreasing threshold in units of the linkage distance. The most robust choice of the threshold value is suggested by the widest range of threshold values in normalized parameter space for which the number of pyramidal neuron types is constant. This choice also indicates two main types of pyramidal neurons in turtle dorsal cortex.

To evaluate the number of pyramidal neuron types within the cellular layer (Fig 1B, layer 2), we analyzed the data set consisting of 225 neurons in a 14-dimensional parameter space using Ward’s linkage method [52]. This bottom-up hierarchical clustering algorithm (Method) generates a linkage plot (a tree diagram called a dendrogram) (Fig 4). The choice of a threshold in this linkage plot then determines the number of distinct clusters (pyramidal neuron types). No rigorous algorithm exists to choose the threshold. One widely used criterion [43, 45, 49] is the Thorndike procedure [53], which holds that the threshold should be drawn at the merge that provides the largest increase in mean intracluster variance. According to this rationale, the unsupervised clustering algorithm identifies two main classes of pyramidal neurons in layer 2 of turtle visual cortex (Fig 4).

**Fig 5:**
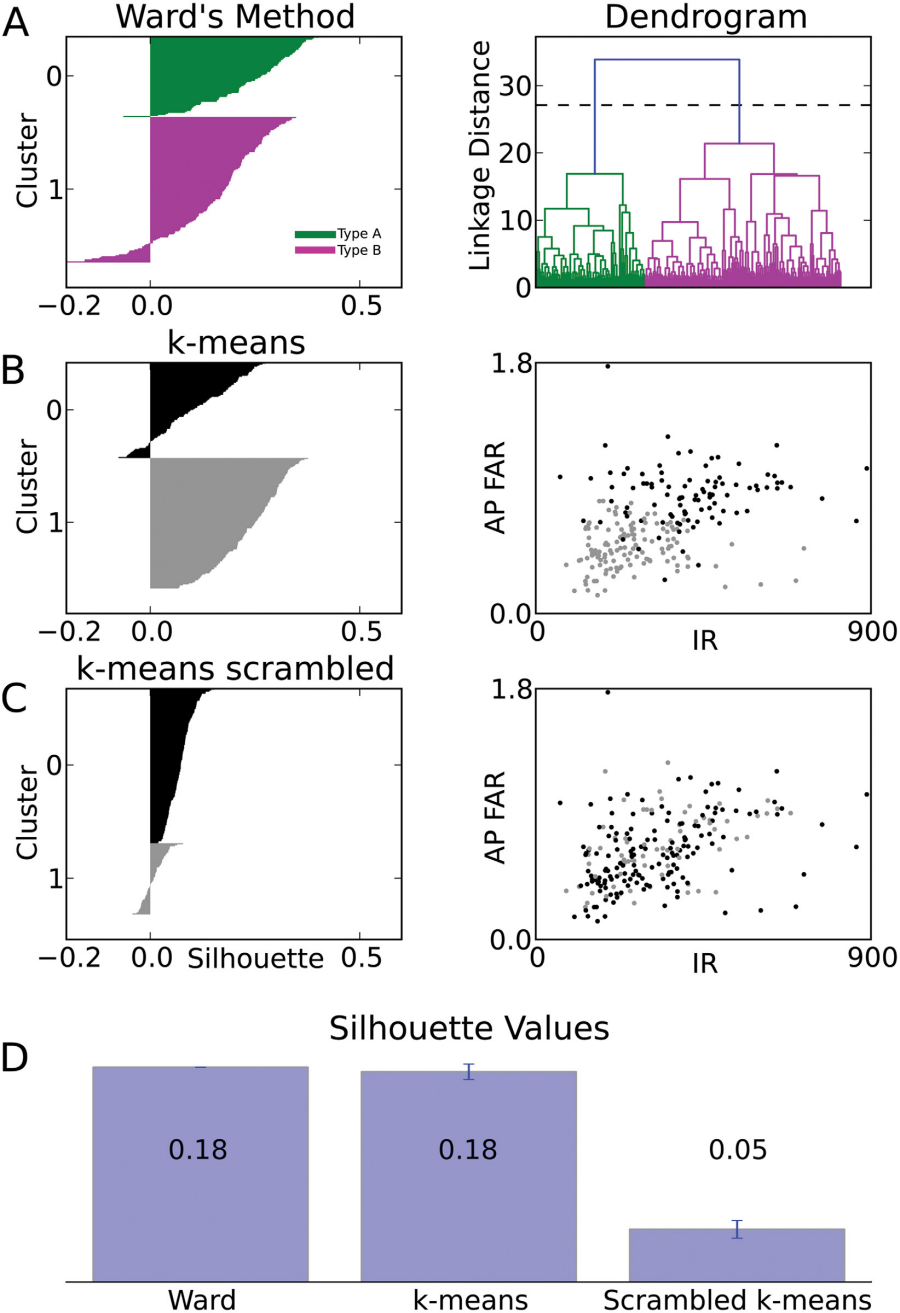
Comparison of clustering algorithms. (A) Silhouette plot of Ward’s clustering. Within each cluster (green/A and magenta/B), cells are ranked (vertical axis) in decreasing order of their silhouette values (horizontal axis). Large positive silhouette values indicate that the data point is close to its cluster’s centroid, whereas negative silhouette values indicate that the data point is closer to the centroid of the other cluster. Right panel: The dendrogram from Ward’s clustering is shown for comparison. (B) Silhouette plot for one rendition of k-means clustering (k = 2). Right panel: The plot of clustered data points (black and gray) within the plane spanned by the input resistance (IR) and the action potential frequency adaptation ratio (AP FAR) illustrates the partial separation of data points from different clusters in this plane alone. (C) Silhouette plot for one rendition of k-means clustering (k = 2) on the scrambled data set. Right panel: The plot of clustered data points (scrambled data set) within the same plane of parameters as in C, reveals the lack of separation caused by scrambling. (D) Comparison between the average silhouette for the Ward’s and k-means (k = 2) clustering of the original dataset and the average silhouette of randomized databases. Scrambling of the data set causes a consistent loss of quality in the clustering. Error bar of the average silhouette for k-means clustering is evaluated by the SD over 1000 renditions of the original data set and by independent randomization for each rendition of the scrambled data sets.

To evaluate the quality of Ward’s clustering, we computed the silhouette values for all neurons [54, 55]. A positive silhouette value indicates that a data point resides closest to its cluster’s centroid, whereas a negative silhouette value indicates that the data point lies closer to the centroid of a different cluster (see Methods), suggesting a possible misclassification. We found that Ward’s clustering of the data set into two clusters results in mostly positive silhouette values (average silhouette <S_ward_> = 0.184), suggesting two cell types within the data (Fig 5A). In contrast, assuming a larger number of clusters (lower threshold) for Ward’s clustering resulted in lower average silhouettes (n = 3: <S> = 0.109) and a greater number of negative individual neuron silhouettes (data not shown).

To evaluate the robustness of the classification into two types, we compared the silhouette values of the bottom-up based Ward’s clustering with those from k-means clustering [56]. The k-means algorithm differs from the Ward’s linkage method in three important ways; it is top-down, contains operational randomness, and predetermines the number of clusters. Applying k-means clustering with k = 2 resulted in mostly positive silhouette values (average silhouette <S> = 0.180) (Fig 5B), thus validating the number of types determined from Ward’s clustering. In contrast, silhouette analysis for k-means clustering when assuming a larger number of types resulted in lower average silhouettes (n = 3: <S> = 0.145) and a greater number of negative individual neuron silhouettes (data not shown).

To evaluate the statistical significance of the k-means clustering of control data, we compared its silhouette values with those from clustering of randomized databases (new database randomization for each k-mean run). Parameter values were shuffled across neurons, destroying the correlations between parameter values while maintaining the same mean, median, and standard deviation of each parameter. We found that the silhouette values for the control data set were consistently higher than the silhouette values for the scrambled dataset (Fig 5C). The analysis, seen in Fig 5, was averaged over 1000 K-means and scrambled K-means classifications and their silhouette values. For quantitative comparison, the average silhouette width was used as a global measure of quality of clustering. We found that both Ward’s clustering (2 types) and k-means clustering (k = 2) of the original data generated significantly larger average silhouette width than k-means clustering (k = 2) on randomized data sets (Fig 5D). This reduction in clustering quality by randomization suggests that the clustering quality of the original database is not generated by accidental random correlations between measurements. Rather, the covariation of properties in the original database indicates the existence of two main types of pyramidal neurons, to which we refer to as type A (green) and B (magenta) in the text and figures.

**Fig 6:**
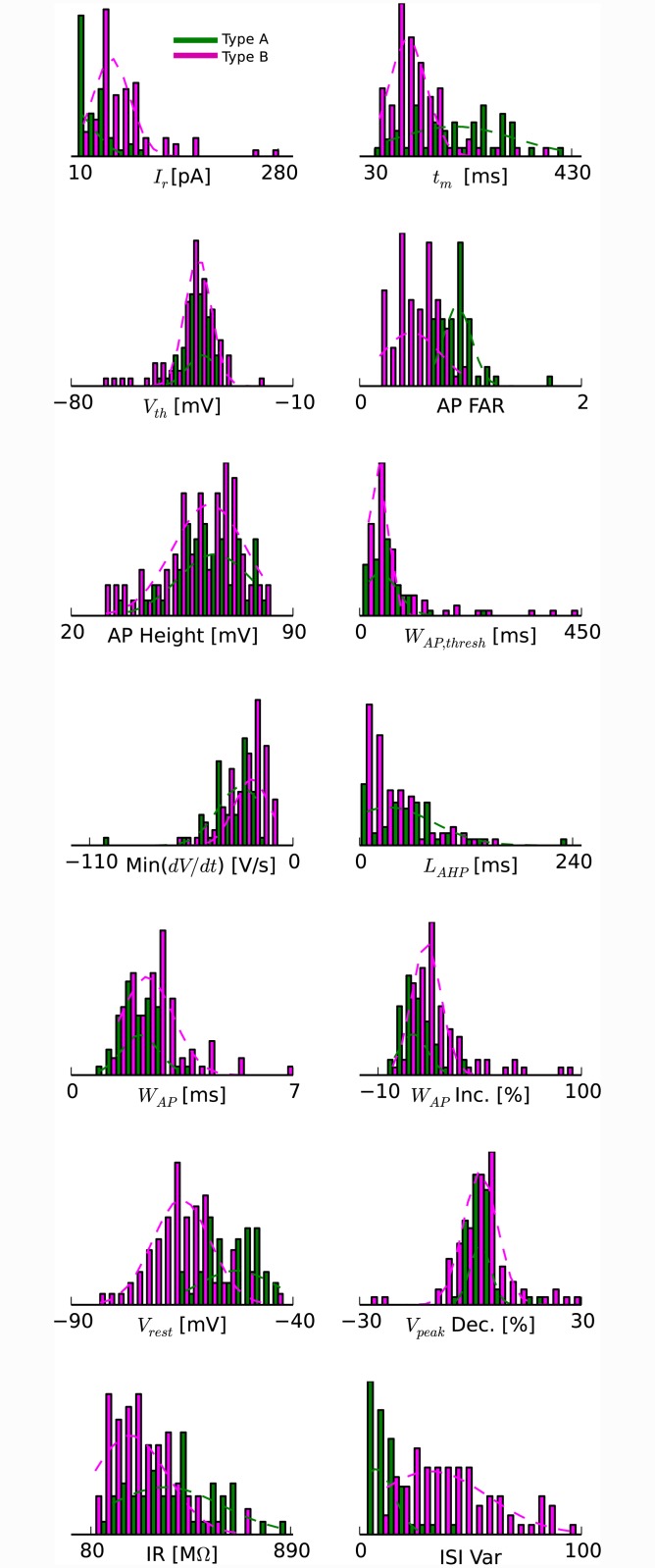
Physiological differences between the two main types of pyramidal neurons based on Ward’s clustering. Histograms of the distribution of the 14 electrophysiological properties shown in Fig 3B and corresponding Gaussian fits for the two main types (green/A and magenta/B). Abbreviations as in Fig 3B.

Plotting the occurrence of parameter values for their respective type assignments generated distributions that resemble Gaussian distributions (Fig 6). This further supports the notion of two types.

**Fig 7:**
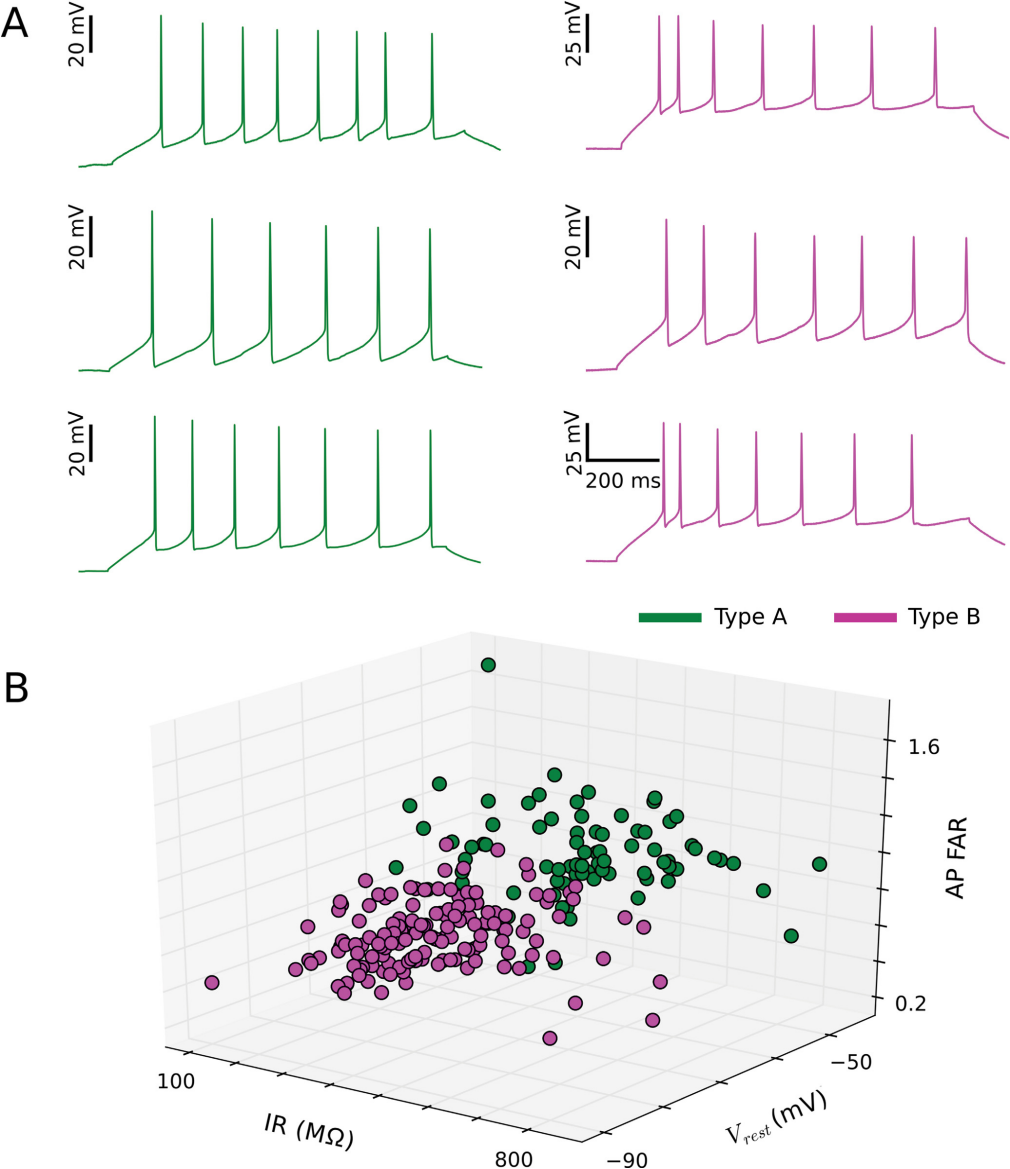
Three physiological parameters produce good separation of the two main pyramidal neuron subtypes. (A) Representative membrane potential responses to somatic current injections for three pyramidal neurons from each subtype (green/A and magenta/B). Input currents ranged from 50 to 70 pA. (B) Partial separation between the two main pyramidal neuron subtypes is observed in a plot of clustered data points (green/A and magenta/B) within the space spanned by the resting membrane potential (V_rest_), the input resistance (IR), and the action potential frequency adaptation ratio (AP FAR).

Of the 14 parameters considered, the two resulting types of pyramidal neurons differed most in three parameters (and their physically related counterpart): resting membrane potential (rheobase current), input resistance (membrane time constant), and action potential frequency adaptation ratio (ISI variability) (Table 1 and Figs 6 and 7). For these three parameters, neither mean was within three standard deviations of the other.

**Table 1:** Properties of the two main types of pyramidal neurons in turtle visual cortex. Mean and standard deviation for all values.

Parameter	Type A	Type B
	(n = 80)	(n = 145)
Rheobase Current (pA):	33.9 ± 13.0	72.6 ± 19.3
AP Voltage Threshold (mV):	-38.9 ± 2.3	-40.2 ± 3.7
AP Height (mV):	64.7 ± 4.8	59.4 ± 5.2
Maximum AP Downslope (mV/ms):	-34.4 ± 8.6	-23.2 ± 5.0
AP half-width (ms):	2.2 ± 0.3	3.1 ± 0.6
Resting Membrane Potential (mV):	-53.2 ± 2.8	-65.8 ± 3.7
Input Resistance (MΩ):	423 ± 80	271 ± 56
AP Frequency Adaptation Ratio:	0.874 ± 0.104	0.499 ± 0.084
AP Duration at Threshold (ms):	59.9 ± 18.0	82.9 ± 47.5
AHP Time to Peak (ms):	56.2 ± 20.5	32.9 ± 15.5
AP Duration Increase (%):	14.6 ± 6.7	23.1 ± 7.7
AP Amplitude Decrement (%):	4.3 ± 3.4	1.0 ± 3.9
ISI Variability:	15.1 ± 5.4	39.8 ± 10.0
Membrane Time Constant (ms):	198 ± 42	121 ± 27

### The impact of network incorporation on cellular individuality

Turtle dorsal cortex consists of approximately 80,000 pyramidal neurons and 20,000 interneurons [21]. A single pyramidal neuron receives some 300 thalamic fiber synapses on the distal portion of its spiny apical dendrites [59], whereas other cortical pyramidal neurons and interneurons contact the thousands of spines along the spatial extent of the apical and basal dendrites [60]. This extensive incorporation of a pyramidal neuron into the cortical circuitry raises the question to what extent the cellular individuality (probed with somatic current injection during ongoing network activity) endures when sensory input pushes the network into a state of high activity [61].

**Fig 8:**
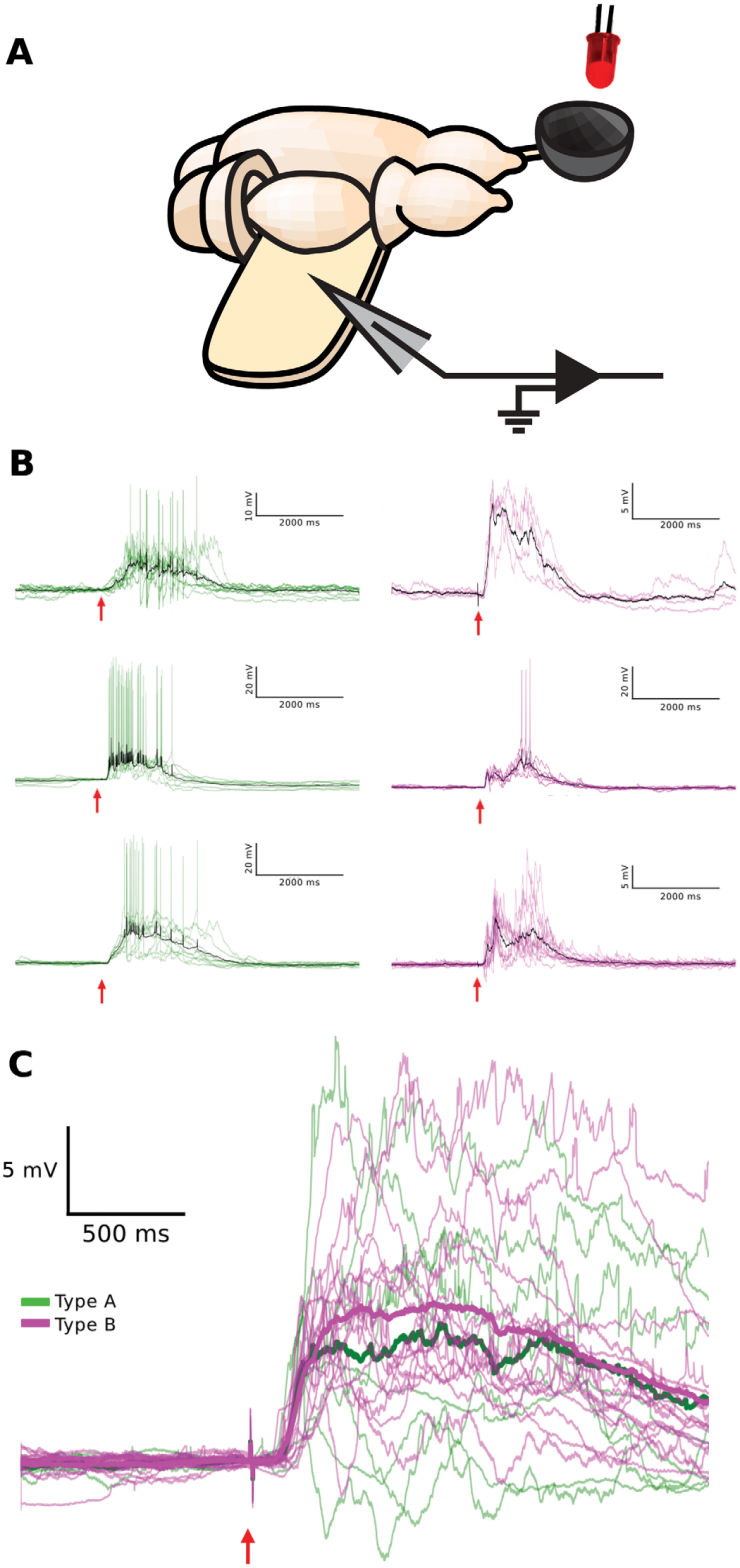
Visual response properties of the two physiologically defined pyramidal neuron types. (A) Schematic of the turtle ex vivo eye-attached whole-brain preparation. A diffuse flash of light from the LED (red) is projected onto the intact retina within the eye-cup (gray bowl), while the membrane potential from a pyramidal neuron is recorded with a patch electrode (gray triangle) inserted into the unfolded visual cortex. (B) Pyramidal neuron membrane potential responses to brief flashes of light (10 ms, 640 nm; red arrow) persist long beyond the duration of the flash, are variable from trial-to-trial, and display sparse spiking. Trial averages are shown in black. Representative membrane potential visual responses to flashes are shown for three pyramidal neurons from each physiologically defined type (green/A and magenta/B). The responses are fluctuating and similar for both types. (C) The time courses of trial-averaged membrane potential (after spike clipping) of all pyramidal neurons recorded in response to flashes (8 type A (green), 16 type B (magenta)). Averages across pyramidal neuron visual responses of the same physiological type are plotted in bold (green/A and magenta/B).

To address this question experimentally, we obtained visually guided whole-cell recordings from pyramidal neurons within the cellular layer of visual cortex using the turtle ex vivo eye-attached whole-brain preparation (Method, Fig 8A). Subsequent to somatic current injection for offline neuron type classification (as described above), we flashed light (640 nm wavelength, LED, 10 ms duration, 30 s wait between trials) onto the spatial extent of the intact retina within the eye cup and recorded the membrane potential visual responses of the cortical pyramidal neuron (Fig 8B). For all pyramidal neurons recorded, visual responses started approximately 100 ms after the brief flash of light and typically lasted for more than 1000 ms. Trial-to-trial variability was extensive, comparable in amplitude to the mean response. The persistent activity and the trial-to-trial variability indicate a significant contribution of the network activity to the cellular visual response of both pyramidal neuron types. Specifically, visual responses consisted of broad depolarization, mediated by a superposition of numerous excitatory and inhibitory postsynaptic potentials. Responses for the two neuron types appeared largely indistinguishable (Fig 8C).

**Fig 9:**
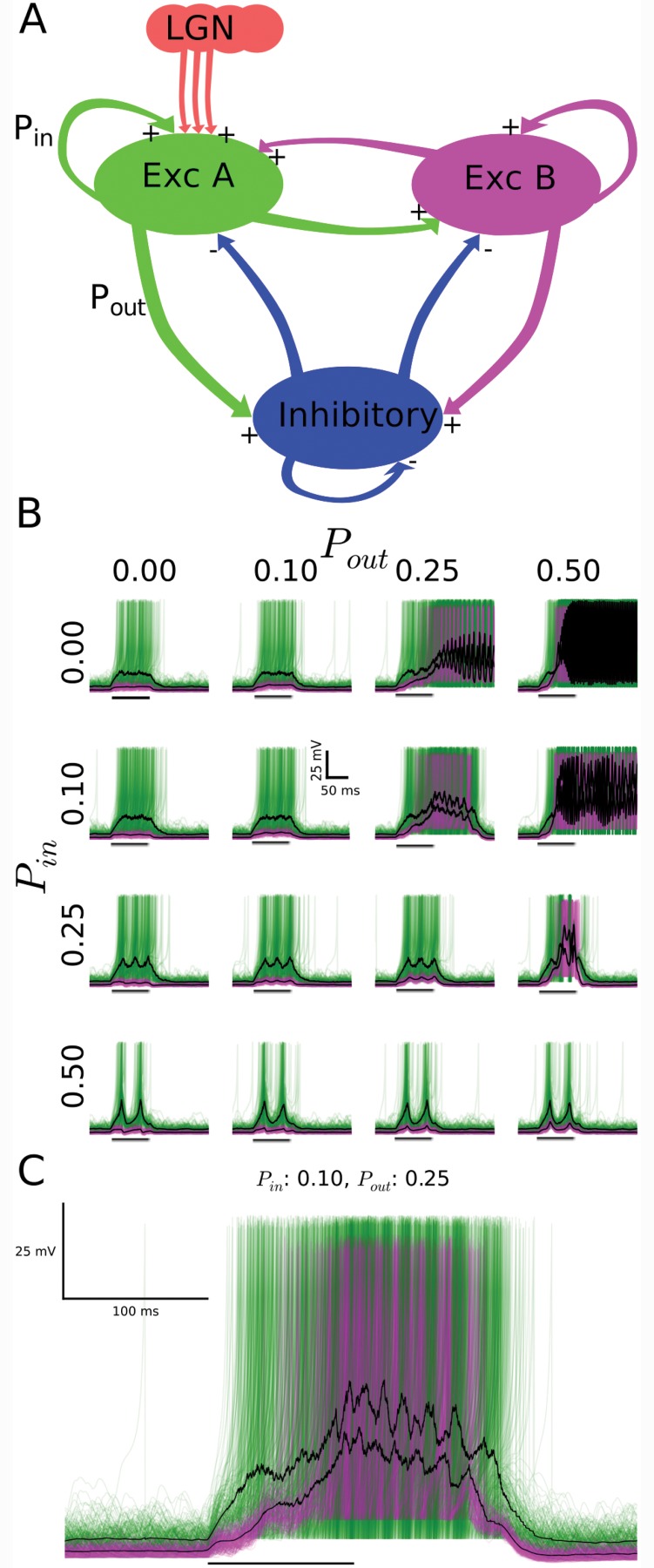
A model network with pulsed external inputs reproduces the similarity of the responses for two types of excitatory neurons. (A) Conceptual cartoon illustrating key model features, including inhibitory neurons (blue) and two types of excitatory neurons (green/A and magenta/B), with excitatory external inputs (“LGN”, orange) limited to one excitatory type (green/A). Intracluster and intercluster connection probabilities are parameterized by P_in_ and P_out_, respectively. (B) Simulated membrane potential responses for the two types of excitatory model neurons (green/A and magenta/B) in response to a brief (100 ms, black horizontal bar) increase in the external input (increased rate of the Poisson pulse trains) for multiple combinations of intracluster and intercluster connection probabilities, each ranging between 0.0 and 0.5. For clarity, simulation results for 200 of the 1000 neurons of each excitatory type are plotted. (C) The connection probability combination of P_in_ = 0.1 and P_out_ = 0.25 best reproduces the experimentally observed persistent activity and the similarity of the type A (green) and the type B (magenta) response to brief external inputs.

The observed similarity of the visual responses for type A and B neurons raises the question to what extent the integration of a cell within a network overrides the contribution of cellular properties to its response. To address this question, we investigated the impact of connectivity on the time course of the response to a brief external input in a model network (Fig 9A). The model network consisted of excitatory model neurons type A and B and of inhibitory model neurons. The three groups of neurons differed in the cellular properties (Table 2). A given neuron from a group projects to neurons within its group and to neurons in the other two groups. The level of connectivity was parameterized by the intracluster (*P*_*in*_) and intercluster (*P*_*out*_) connection probabilities. To increase the model challenge of reproducing similar responses with different neuron types, we connected external inputs exclusively to model neurons type A. This differential external input was further motivated by experimental evidence for afferent inputs to one type only from (i) earlier molecularbiological investigations in turtle [31], and (ii) studies of piriform cortex [23].

**Table 2:** Parameters used to define the model neurons.

Parameter	Excitatory A (visual input)	Excitatory B(no visual input)	Inhibitory
a	0.02	0.02	0.1
b	0.25	0.2	2
c	-65	-65	-50
d	0.05	8	2
C_m (μF)	20	30	20
R (MΩ)	400	250	100
N	500	500	150

We investigated the membrane potential of excitatory model neurons type A and B in response to a brief increase in the spike rate of the external inputs for varying levels of connectivity. For vanishing intercluster connectivity, the response was limited to type A, as expected (Fig 9B). With increasing intercluster connectivity, the response in type A started to activate the type B and inhibitory neurons, resulting in complex network activity. Importantly, at an intermediate level of connectivity (*P*_*in*_ = 0.1; *P*_*out*_ = 0.25), the simulated model responses (Fig 9C) resembled qualitatively the time course and similarity of the recorded visual responses of the two types of pyramidal neurons in turtle visual cortex (Fig 8B).

## Discussion

The dichotomy between cellular individuality and network integration raises a profound question in neuroscience. To what extent does subtype identity of a pyramidal neuron impact the neuron’s dynamic and signal processing when it is incorporated in an extensively interconnected network, such as microcircuits of cerebral cortex? To address this question, we examined the classification of turtle visual cortex pyramidal neurons based on intrinsic electrophysiological properties (Fig 6). We then probed the neuron’s responses to visual stimulation (Fig 8), which concurrently pushed the network into a state of intense emergent activity. We discovered two main electrophysiological types of pyramidal neurons (Fig 4) and found that their visual responses were indistinguishable and apparently dominated by emergent network activity (Fig 8B and 8C). Given the limitation in our study of visual stimulation to diffuse flashes, we cannot exclude the possibility that exploration of more complex stimuli would reveal cell-type specific visual responses. A model network, when tuned to a suitable level of connectivity, reproduced the similarity of the responses of the two cell types (Fig 9). In future model investigations it will be fruitful to investigate in an extensive parameter search under what conditions of intrinsic physiology and connectivity cell-type specific differences in the responses to external inputs can arise.

### Parameter selection

In this investigation of pyramidal neuron types, parameter selection was guided by minimizing redundant information and by evidence from previous studies that parameter values varied significantly among subpopulations of pyramidal neurons. For instance, the following parameters have previously been shown to vary between subgroups of pyramidal neurons in neocortex: input resistance [37, 38, 40, 42]; resting membrane potential [38]; membrane time constant [39]; action potential amplitude [37, 47], voltage threshold [37], duration at half-maximum amplitude [38, 40], duration at threshold [39, 42], maximum rate of decay [40], rheobase current [40], the time to the peak of the afterhyperpolarization [39, 42]; action potential frequency adaptation ratio [37, 50]; action potential duration increase and action potential amplitude reduction [43, 49]. In addition, resting membrane potential, input resistance, action potential frequency adaptation ratio, and rheobase current have all also been shown to vary with genetically defined subtypes among pyramidal neurons in mouse visual cortex and somatosensory cortex [62].

### Classification algorithms

After feeding measured parameters into the Ward’s linkage method clustering algorithm, the choice of the threshold intergroup linkage distance determines the number of inferred clusters (Fig 4). The subjectivity of this choice has been addressed using different strategies. One strategy is to reduce the subjectivity inherent to the choice of threshold by adding cells of different types (e.g. pyramidal neurons in an interneuronal classification study) to the data base [43, 49, 55, 63] or by overlaying morphology on purely electrophysiological clusters [45, 46, 64]. Another strategy is to elevate the confidence levels behind clustering by analyzing the clustering results for different threshold levels and comparing relative “accuracy” measurements across those schemes. Strategies to this end include silhouette analysis [54, 55] and the Mann-Whitney Test [38, 65]. A third family of strategies uses inherent properties of the clustering itself to determine the threshold. With each intercluster merge the number of clusters decreases but the mean intracluster variance increases. The Thorndike procedure [53] suggests that the threshold should be drawn at the merge that provides the largest increase in mean intracluster variance and has been used in several neuronal classification studies [43, 45, 49]. The application of fuzzy clustering algorithms to the classification of fusiform neocortical neurons suggested a final test [66]. For each number of subtypes from N = 1 (all cells are indistinct and members of one superpopulation) to N = the number of cells recorded (all cells are unique and no meaningful crossover exists among them) there is some finite range of threshold linkage distances for which N subtypes appear from the data. The correct N, and from that the correct threshold, should be chosen from the largest range of thresholds which gives the same number of subtypes (Fig 4). For the study presented here, the number of subtypes was determined by applying the Thorndike and Battaglia criteria and was corroborated through k-means [56] and silhouette [54] analysis.

### Two pyramidal neuron types in allocortex

The allocortex is a phylogenetically ancient trilaminar cortical structure [15]. Well-studied contemporary model systems of allocortex are the mammalian piriform cortex [19, 67] and hippocampus [20] and the reptilian dorsal cortex [21]. All three model systems are largely congruent in their microcircuit structure [17, 18]. Layer 2 contains densely packed somata of pyramidal neurons. Pyramidal neuron dendrites and axons project into the adjacent layers 1 and 3. Afferents make en-passant synapses in superficial layer 1 on interneurons and on distal segments of dendrites from layer 2 pyramidal neurons. Scattered interneurons in layer 1 and 3 mediate feed-forward and feed-back inhibition (Fig 1B).

The laminar specificity of pyramidal neuron types in the six-layered neocortex raises the question whether the sole layer of pyramidal neuron somata in allocortex consists of discrete types of pyramidal neurons. Based on morphological and intrinsic electrophysiological properties, two types of pyramidal neurons have been identified in mouse piriform cortex [22, 23] and rat hippocampus [24].

In this study, we have extended the question to a third model system of allocortex, namely the dorsal cortex of turtle. Earlier investigations of pyramidal neurons in this system revealed a variation of input resistance [48], firing patterns [29], axonal projection targets [30], and molecular markers [31]. However, these valuable studies did not quantify to what extent the variation of those properties reflects the existence of pyramidal neuron subtypes or the broad distribution of properties in one neuron type. Here, based on the parameterization of intrinsic electrophysiological properties and unsupervised clustering, we have identified two types of pyramidal neurons in turtle dorsal cortex. Broadly speaking, type A neurons tend to be more excitable and tend to show less spike adaptation than type B neurons (Figs 6 and 7).

### Type-specific connectivity

The apparent congruence of microcircuit structure and the coincidence of two types of pyramidal neurons in the three model systems of allocortex raises the question to what extent neuronal type correlates with connectivity in the three systems. In mouse hippocampus, the two distinct principal neuron types in layer 2 are inversely modulated by glutamate and acetylcholine acting on metabotropic receptors, which advances the notion that the two types support two parallel signal pathways [24]. In mouse piriform cortex, the semilunar principal neurons in layer 2 receive stronger afferent inputs, whereas the superficial pyramidal neurons of the same layer receive stronger associational (intracortical) inputs [23]. Our visual response data from the two types of pyramidal neurons in turtle dorsal cortex, combined with the model investigation, suggest that strong associational (intracortical) inputs are common to both types.

### Comparative analysis of allocortex and neocortex

Given the ancestral position of allocortex in evolutionary history [16], it is instructive to highlight our results from turtle dorsal cortex within the context of related studies of pyramidal neuron subtypes in neocortex. First, of the 14 parameters considered for the cluster analysis, the two resulting types of pyramidal neurons differed most in their excitability (resting membrane potential, rheobase current) and spike adaptation (spike frequency adaptation ratio, ISI variability). Interestingly, those parameters have previously been shown to vary across genetically defined subtypes of layer 5 pyramidal neurons in mammalian somatosensory and visual cortex [62]. Second, the separation of early and late sensory responses, visible in some neurons, is not unique to turtle visual cortex, rather they have previously been observed in mouse barrel cortex [68]. Third, the similarity of the fluctuating responses for the two types of pyramidal neurons in turtle visual cortex resembles the previously observed similarity of receptive field properties for morphological and electrophysiological pyramidal neuron subtypes within the same layer of cat visual cortex [46]. Fourth, the inference of two pyramidal neuron subtypes in turtle visual cortex is consistent with the molecular evidence for cortical L4/input and L5/output cell-type homologies across amniotes [31]. It is thus tempting to speculate that the classified type A and B pyramidal neurons of turtle visual cortex express two selective mRNA profiles that match the mammalian cortical L4/input and L5/output neurons, respectively. However, tests of this tantalizing speculation must await future studies.

### The dichotomy of cellular individuality and associational circuits

A subtype-specific division of labor is believed to create a rich substrate for computation [69, 70]. On the other hand, profuse associational connections are thought to implement complex sensory processing [71]. Here we showed that pyramidal neuron membrane potential responses to a diffuse brief flash of light were characterized by persistent activity of high trial-to-trial variability. The response was not type-specific (Fig 8). This observation, combined with the model investigation (Fig 9), suggests that the answer to the signal-processing role of neurons vs network appears to depend on the question/perspective at hand. This neuron-network duality of circuit dynamics and computation addresses the inability of the classical concepts “neuron” or “network” to describe the dynamics and computation of microcircuit-scale cortical tissue during the visual processing of spatiotemporal complex scenes.
